# Decoration of Poly-3-methyl Aniline with As(III) Oxide and Hydroxide as an Effective Photoelectrode for Electroanalytical Photon Sensing with Photodiode-like Behavior

**DOI:** 10.3390/mi14081573

**Published:** 2023-08-09

**Authors:** Mohamed Rabia, Asmaa M. Elsayed, Maha Abdallah Alnuwaiser

**Affiliations:** 1 Nanomaterials Science Research Laboratory, Chemistry Department, Faculty of Science, Beni-Suef University, Beni-Suef 62514, Egypt; mohamedchem@science.bsu.edu.eg; 2 TH-PPM Group, Physics Department, Faculty of Science, Beni-Suef University, Beni-Suef 62514, Egypt; 3 Department of Chemistry, College of Science, Princess Nourah bint Abdulrahman University, P.O. Box 84428, Riyadh 11671, Saudi Arabia

**Keywords:** poly-3-methyl aniline, As_2_O_3_–As(OH)_3_, photodetector, photodiode, photon sensitivity

## Abstract

This study achieved the decoration of poly-3-methyl aniline (P3MA) with As_2_O_3_–As(OH)_3_ using K_2_S_2_O_8_ and NaAsO_2_ on the 3-methyl aniline monomer. This resulted in a highly porous nanocomposite polymer composite with wide absorption optical behavior, an average crystalline size of 22 nm, and a 1.73 eV bandgap. The photoelectrode exhibited a great electrical response for electroanalytical applications, such as photon sensing and photodiodes, with a J_ph_ of 0.015 mA/cm^2^ and J_o_ of 0.004 mA/cm^2^. The variable J_ph_ values ranged from 0.015 to 0.010 mA/cm^2^ under various monochromatic filters from 340 to 730 nm, which demonstrates high sensitivity to wavelengths. Effective photon numbers were calculated to be 8.0 × 10^21^ and 5.6 × 10^21^ photons/s for these wavelength values, and the photoresponsivity (R) values were 0.16 and 0.10 mA/W, respectively. These high sensitivities make the nanocomposite material a promising candidate for use in photodetectors and photodiodes, with potential for commercial applications in highly technological systems and devices. Additionally, the material opens up possibilities for the development of photodiodes using n- and p-type materials.

## 1. Introduction

The relationship between energy and photon sensing is considered at its highest level under the great reduction in nonrenewable energy sources and the need for renewable resources. Under the rapid development of the industrial revolution, the demand for energy increased dramatically, the population increased, and the need for light sensing increased because of vital human activities [1,2]. The demand for friendly and renewable energy has remained a priority for industrialized countries, which has led to the development of many technologies, such as solar energy controlled through the use of photon sensors. Photon intensity and wavelength detection are applied in energy fields such as solar-powered photovoltaic scattering of water for proton (hydrogen) production and low carbon dioxide for fuel production, which has become a significant research field due to the promise of solar energy harvesting and storage in the form of chemical energy. A new alternative to photoelectrode systems inside complex technological systems for the detection and control of light in a wide range of optical regions depends on the optical properties of the nanomaterials/semiconductors [3,4]. III–V semiconductors are commonly used for efficient photovoltaic applications because of their direct beam vacuum, high absorbance of sunlight, high electron mobility, and controlled crystal evolution [5,6,7]. Under light radiation, photoinduced electrons and holes represent the sensitivity of nanomaterials to photon detection [8,9]. 

Another way to increase electrode efficiency is to use materials with great morphologies to capture light, in which most of nanoscale materials with porous morphologies have great capability for light sensing with high detectivity [10,11]. Moreover, one of the main reasons for using photovoltaic detection materials is to reduce the total production cost. From semiconducting inorganic compounds (groups III–V, Si, etc.), transition element oxides (BiVO_4_, Fe_2_O_3_, TiO_2_, and Cu_2_O), sulfides (NiS and CdS), acacinitrides (BaNbO_2_N), and chalcogenides that are prepared under controlled nanoscale morphologies are the most used materials for this purpose [12,13,14,15]. 

The preparation of photoelectrodes from polymers is less complex and more suitable for the production of large-scale devices than electrodes based on inorganic materials only. A distinctive property of polymers is that the positions of the bonds can be easily adjusted, unlike inorganic ones. Moreover, polymeric photoelectrodes have high optical absorption. Recently, metal oxide/polymer nanocomposites have demonstrated promising behavior for light detection due to its attractive photocatalytic properties, which combine the optical properties of the composite materials [16].

As_2_O_3_ is indeed a highly stable oxide that is recommended for application within various devices involved in light sensing and current control. Its exceptional stability allows it to exhibit light absorbance across a wide optical region, enabling the capture of a significant number of photons within this range [17]. This absorbance property makes As_2_O_3_ suitable for capturing and detecting energy photons, thus making it ideal for a range of optoelectronic applications and energy conversion development [18].

The high light absorbance exhibited by As_2_O_3_ when subjected to incident illumination further enhances its light-sensing capabilities. This characteristic is expected to enables the material to effectively sense and measure the number of photons in different optical regions during its application as a photoelectrode. Consequently, As_2_O_3_ demonstrates a fast response to light, allowing for quick detection and response times, which is advantageous for applications that require rapid light capture.

While As_2_O_3_ exhibits semiconducting properties, it is expected to have a small or negligible dark current (J_o_) value. This implies that the ratio of the desired signal to unwanted noise, known as the signal-to-noise ratio, is highly promising in photodetectors based on As_2_O_3_. The low dark current contributes to improved sensitivity and ensures that the detected light signal stands out prominently against background noise.

Combining these advantages, including light sensitivity, stability, and cost-effectiveness, As_2_O_3_ has emerged as a highly promising material for use in optoelectronic systems [17]. Its ability to efficiently capture and sense light, coupled with its low cost and stable characteristics, positions it as a favorable candidate for a wide range of applications in optoelectronics, where precise light sensing and energy conversion are vital. Ongoing research and development continue to explore and optimize the use of As_2_O_3_ in optoelectronic systems, further expanding its potential for various applications.

Recent studies have attempted to improve the efficiency of photodetector and photodiode devices that are evaluated by electrical measurements, but the achieved J_ph_ values still have significant drawbacks. For example, Bai et al. [19] achieved 107 µA with a ZnO/CuO nanocomposite, while Costas et al. [20] achieved only 0.1 nA. Graphene/P3HT was also investigated in previous studies [21], but the achieved J_ph_ values were too limited for practical photodetector applications.

In this context, a new photoelectrode made of P3MA decorated with As_2_O_3_–As(OH)_3_ was developed and analyzed for its potential as a photodetector and photodiode. The J_ph_ and Jo values were estimated under various monochromatic light and dark/light conditions, demonstrating the efficiency (R) and detectivity (D) of the photoelectrode. Additionally, the linear dynamic range was calculated, indicating the ability of the photoelectrode to convert photons into current. Overall, this photoelectrode shows great promise for optical applications due to its high response to incident photons.

## 2. Experimental Section

### 2.1. Materials and Characterization

Sodium arsenate (NaAsO_2_) and 3-methyl aniline were purchased from Merck Co., Ltd., Darmstadt, Germany. K_2_S_2_O_8_ was obtained from Pio-Chem Co., Egypt. Dimethylformamide was sourced from Sigma-Aldrich Co., Ltd., St. Louis, MO, USA.

The nanomaterials are characterized using several techniques to study their properties thoroughly. TEM (JEOL, Tokyo, Japan) was used to obtain 2D images, while SEM (ZEISS, Jena, Germany) was used to obtain 3D topography. XRD (PANalytical Pro, Waltham, MA, USA) was used to determine the peak position at 2-theta, and XPS (Kratos Aanl, London, UK) was used to determine the electron volt positions for the peaks through the photoelectron for elemental determination. FTIR (Bruker, Billerica, MA, USA) was used to confirm the functional groups, while the optical properties were observed using a Birkin Elmer spectrophotometer to measure the absorbance behavior.

### 2.2. P3MA Preparation

The P3MA was prepared by oxidatively polymerizing 3-methyl aniline with K_2_S_2_O_8_. The monomer and oxidant were present at concentrations of 0.12 and 0.15 M, respectively. An acid medium and dopant were used during the polymerization, which was achieved using 0.5 M HCl. The HCl was used to dissolve the polymer and improve its conductivity.

### 2.3. As_2_O_3_–As(OH)_3_/P3MA Optoelectronic and Photodiode Preparation

The As_2_O_3_–As(OH)_3_/P3MA nanocomposite was prepared by carrying out the oxidative polymerization of 3-methyl aniline with 0.07 M K_2_S_2_O_8_ in the presence of 0.15 M NaAsO_2_ at room temperature. This led to the formation of a dark green polymer film on glass, which was then purified through centrifugation to obtain the As_2_O_3_/P3MA nanocomposite.

In our photodiode preparation, we utilized a previously prepared thin film of polypyrrole (Ppy) [22]. This Ppy thin film was deposited as a p-type material. Subsequently, we deposited a thin film of the As_2_O_3_–As(OH)_3_/P3MA nanocomposite, which served as an n-type material in the photodiode structure. This combination of materials allowed for the formation of an efficient photodiode with complementary p–n junction characteristics, paraphrasing.

### 2.4. The Electrical Study

The electrical study of the As_2_O_3_–As(OH)_3_/P3MA nanocomposite film for electro-analytical photon detection under various light conditions or wavelengths was carried out using the CHI608E device. The current density in light (J_ph_) or dark (J_o_) was evaluated, and the As_2_O_3_/P3MA nanocomposite film was contacted on both sides with the CHI device through silver paste. The efficacy of the prepared film to the light was evaluated using a metal halide lamp as a photon source, and the photon sensitivity was considered the main factor. The incidence light wavelengths or intensities were well controlled using an optical filter.

## 3. Results and Discussion

### 3.1. Analyses

**Figure 1:**
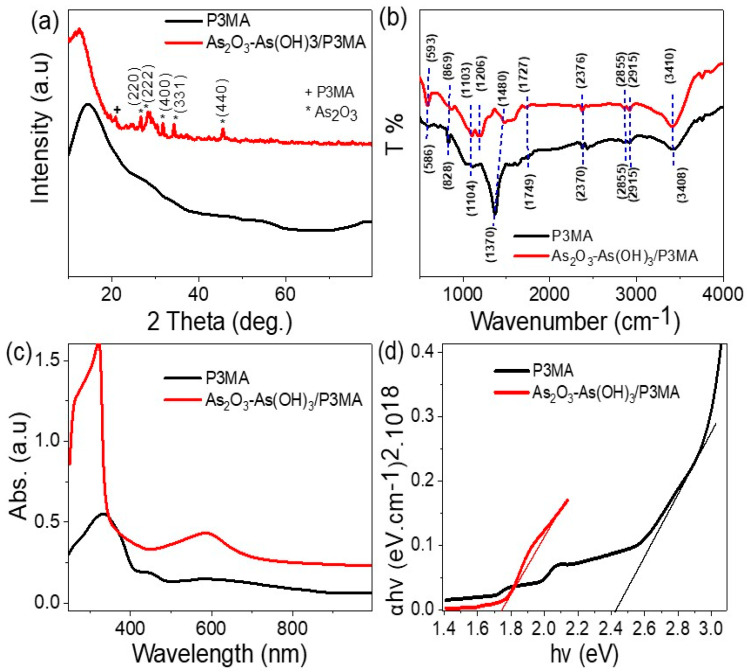
The characterization analyses of P3MA (black curve) and the As_2_O_3_–As(OH)_3_/P3MA composite (red curve) through (**a**) XRD, (**b**) FTIR, (**c**) optical absorbance, and (**d**) the calculated band gap.

The XRD analysis of the P3MA and As_2_O_3_–As(OH)_3_/P3MA nanocomposite is shown in Figure 1a. The P3MA exhibited amorphous behavior, as evidenced by the broad peaks observed. In contrast, the As_2_O_3_–As(OH)_3_/P3MA composite showed sharp peaks, indicating the formation of crystalline structures of both P3MA and inorganic materials (As_2_O_3_–As(OH)_3_). The peak observed at 21.0° for P3MA indicates that the polymer exhibited additional crystalline behavior after the composite formation. Furthermore, the peaks observed at 26.6°, 28.4°, 31.8°, 34.5°, and 45.5° in the growth directions (220), (222), (400), (311), and (440), respectively, are attributed to the As_2_O_3_ material [23]. However, the XRD analyses did not reveal much about As(OH)_3_, as these materials typically exhibit amorphous behavior [24,25]. The crystalline size (D) of this composite was evaluated using Scherrer’s equation (Equation (1)) by considering the highest peaks at the 331-growth direction (2-theta, Bragg angle = 34.5°) and the half maximum of the peak (W). Based on this calculation, the D value is 22 nm.

D = 0.9λ/W cosθ(1)

The FTIR spectroscopy in Figure 1b was used to identify the materials based on their band positions in cm^−1^. For P3MA (black curve), the bands at 3408, 1727, 1480, and 1206 cm^−1^ correspond to the N–H, C–C, C=C, and C–N groups, respectively [26]. The bands at 1103 and 869 cm^−1^ are characteristic of C–H groups. The composite As_2_O_3_–As(OH)_3_/P3MA (red curve) showed similar bands to those of P3MA, but with some red shifts, indicating the interaction with As_2_O_3_–As(OH)_3_ [27,28,29]. Moreover, a noticeable enhancement was observed in the peak at 3410 cm^−1^ for O–H groups, alongside the N–H groups of the polymer.

The optical behavior of P3MA (black curve) and the As_2_O_3_–As(OH)_3_/P3MA composite (red curve) was analyzed, as shown in Figure 1c. The addition of As_2_O_3_–As(OH)_3_ to P3MA caused a significant increase in optical absorbance, which was observed in the intensities of the peaks and the coverage of the optical regions from UV to near IR spectra. This indicates that the composite has a wide photon-sensing ability that affects the electron transition in the optical regions of UV and visible light. Based on this optical behavior, the composite has potential for use in light sensing and various optical applications across a wide spectrum.

Using the theoretical Tauc equation (Equation (2)) [22,30,31], which relates the absorption coefficient of a material to the photon energy, the bandgap energy (E_g_) can be demonstrated using α and ν, which is the absorption coefficient and frequency correspondingly (Figure 1d):

(αhν)^0.5^ = A(hν − E_g_) (2)

From this equation and figure, the estimated E_g_ is 2.42 and 1.73 eV for P3MA and the As_2_O_3_–As(OH)_3_/P3MA composite, respectively.

This reduction in the bandgap value for the composite indicates the increase in the photon absorption ability of the composite. This also suggests that the insertion of As_2_O_3_–As(OH)_3_ into P3MA provided a good charge transfer from the inorganic materials to the polymer matrix. The bandgap reduction also indicates the suitability of the composite for applications related to optoelectronics: Solar cells, photocatalysts, and optical sensors [32,33].

**Figure 2:**
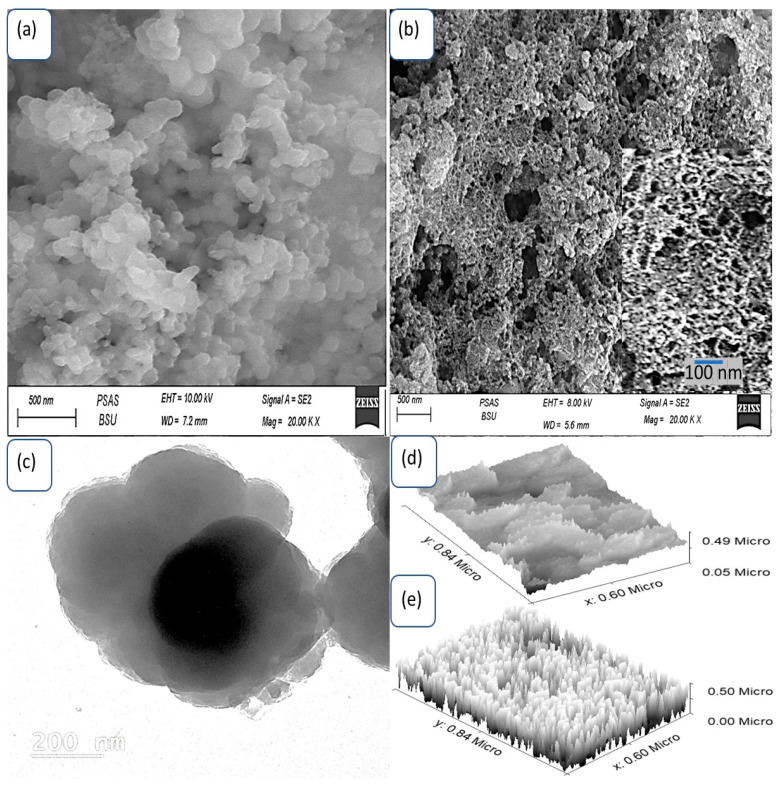
The SEM morphology of (**a**) P3MA and (**b**) the As_2_O_3_–As(OH)_3_/P3MA nanocomposite (with inserted magnified image). (**c**) TEM of the As_2_O_3_–As(OH)_3_/P3MA composite. Cross-section and roughness of (**d**) P3MA and (**e**) the As_2_O_3_–As(OH)_3_/P3MA nanocomposite.

The topography of both P3MA and the As_2_O_3_–As(OH)_3_/P3MA nanocomposite was characterized using SEM, TEM, and theoretical roughness estimation, as shown in Figure 2. SEM analysis revealed a noticeable difference in morphology between P3MA and As_2_O_3_–As(OH)_3_/P3MA. P3MA exhibited nonuniform particles with an average length of 100 nm, whereas the As_2_O_3_–As(OH)_3_/P3MA nanocomposite displayed a well-connected network of fine particles forming a porous structure. Each particle in the nanocomposite had an average length of 20 nm, and there were instances of particle agglomeration, forming larger particles (300 nm). The TEM image in Figure 2c further confirm this observation, with the dark regions indicating the presence of the inorganic material As_2_O_3_–As(OH)_3_ and the faint regions representing the polymer matrix in which these particles were embedded.

The surface cross-section and roughness are evaluated theoretically with the Gwydion program. The nonuniform shape of P3MA was evident in the roughness estimation, while the highly distinct particles of the As_2_O_3_–As(OH)_3_/P3MA nanocomposite were clearly identified.

**Figure 3:**
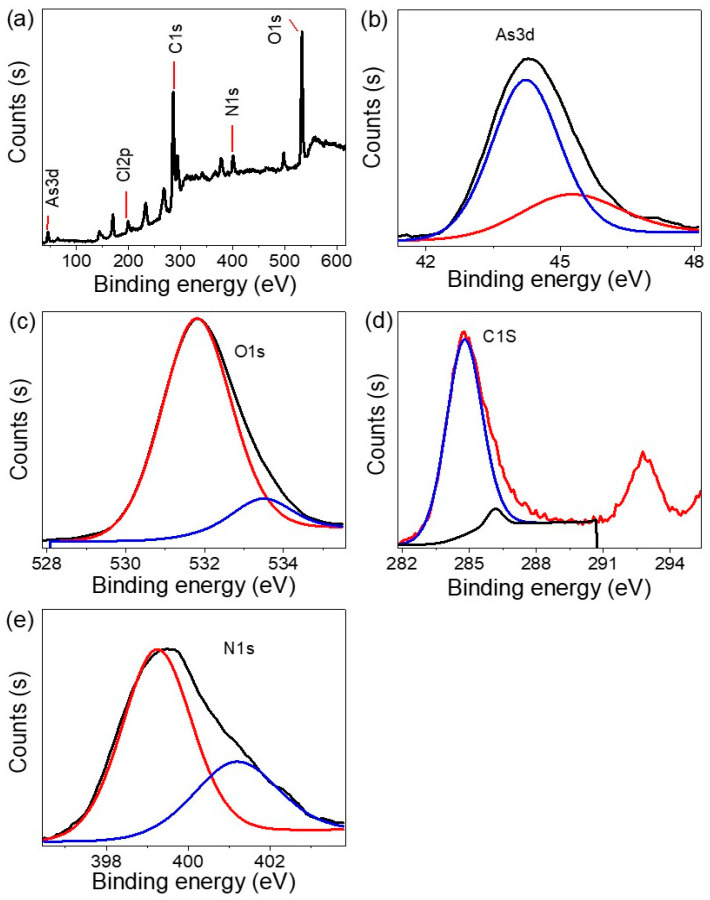
The XPS survey of (**a**) the As_2_O_3_–As(OH)_3_/P3MA nanocomposite. The XPS of the individual elements (**b**) As (**c**) O, (**d**) C, and (**e**) N spectra.

Chemical analysis of the As_2_O_3_–As(OH)_3_//P3MA nanocomposite was performed using XPS, and the results are presented in Figure 3a for the overall chemical survey and in Figure 3b specifically for the As3d spectra. The As3d spectrum showed a peak position at 45 eV, indicating the presence of As(III) in the nanocomposite. The O1s spectrum appeared at 532 eV, confirming the presence of oxygen in the composite as illustrated in Figure 3c. The C and N 1s spectra, representing the elements related to the polymer, were observed at 285 and 400 eV, respectively, as demonstrated in Figure 3d,e, respectively. Additionally, the presence of the Cl element from the HCl acid used during the synthesis was detected at 199 eV. These XPS results provide evidence for the formation of the As_2_O_3_–As(OH)_3_//P3MA nanocomposite, with the presence of As(III) and other relevant elements.

### 3.2. Electrical Measurements

The As_2_O_3_–As(OH)_3_/P3MA composite has been shown to have promising optical properties that make it suitable for use in photodetectors, particularly for detecting light intensity using electroanalytical photon intensity determination. This process involves studying the current–voltage relationship, where the current density (J_ph_) is observed under light or an optical filter and compared to the current density (J_o_) in the dark. The difference between these values indicates the potential application of the photodiode, which involves electron transfer between n- and p-type materials. CHI608E is used for all of these electrical studies, which applies potential and registers the produced current values through the photo-generated carriers that contribute to the total current. Halide lamps serve as the photon sources for these experiments.

**Figure 4:**
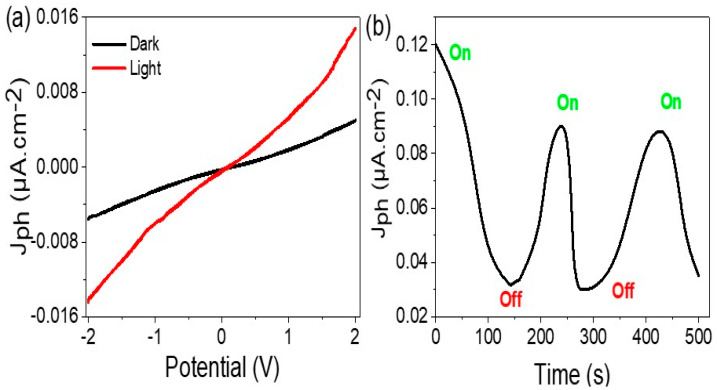
(**a**) The electrical study of the As_2_O_3_/As(OH)_3_/P3MA nanocomposite film through current–voltage under dark and light conditions and (**b**) the J_ph_ value at 2.0 V.

The results of the electrical measurements demonstrate that the As_2_O_3_–As(OH)_3_/P3MA composite has a high response to photons, as indicated by the significant difference between the J_ph_ (0.015 mA.cm^−2^) and J_o_ (0.004 mA.cm^−2^) values under light and dark conditions, respectively (Figure 4a). This behavior is for the generated hot electrons upon photon absorption and clouded on the active sites of the material and provides a high J_ph_ value (at a voltage bias of 2.0 V). The low J_o_ value reflects that the material can prevent current flow in the dark and generate electricity in the circuit in the presence of light, recommending this material as a promising candidate for photodiode applications. The on/off chopped light experiment further demonstrates the sensitivity of the material to light and the corresponding change in J_ph_ values (Figure 4b). Therefore, the J_ph_ value can be used to evaluate the sensitivity or efficiency of the material for photon capture and detection.

**Figure 5:**
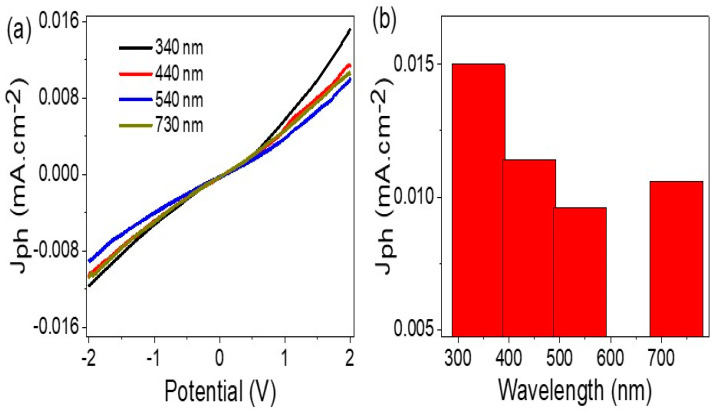
(**a**) The electrical study of the As_2_O_3_/As(OH)_3_/P3MA nanocomposite photoelectrode through current–voltage under various monochromatic light conditions (340–730 nm) and (**b**) the J_ph_ value at 2.0 V.

The As_2_O_3_/As(OH)_3_/P3MA nanocomposite film photoelectrode demonstrated high sensitivity to incident light intensity and wavelength, as shown in Figure 5a,b. The current–voltage relationship in Figure 5a shows that photosensitivity increases as the incidence light wavelengths decreased from 730 to 340 nm, which correlates to an increase in the produced J_ph_ values. The small J_ph_ value of 1.73 eV, calculated previously from Figure 1d, is promising and smaller than the normal visible bandgap from 1.95 to 3.26 eV. This indicates that the photodetector or photodiode device has the ability to sense light through the electron transition from the lower to the upper conducting level.

The bandgap value of the As_2_O_3_/As(OH)_3_/P3MA nanocomposite is considered promising for the efficient utilization of sunlight in the Vis and IR regions. The large percentage of sunlight present in these regions makes the nanocomposite well suited for capturing solar energy. Researchers in the field aim to achieve an optimal device system by carefully controlling the bandgap value of the nanocomposite.

One notable advantage of the As_2_O_3_/As(OH)_3_/P3MA nanocomposite is its cost-effectiveness and suitability for mass production. Its preparation on normal slide glass further contributes to its affordability. This low-cost nature of the device makes it appealing for both technical and commercial applications. It can function as a photodetector or photodiode, enabling the detection of light and control of current flow.

This dual advantage of the As_2_O_3_/As(OH)_3_/P3MA nanocomposite poses a significant challenge for many researchers in the field. They strive to design and optimize devices that possess both light detection capabilities and the ability to regulate current flow. Meeting this challenge opens up opportunities for various applications, ranging from renewable energy harvesting to sensing and optoelectronic systems.

The technical and commercial viability of a low-cost, high-performance device that can detect light and regulate current flow is a desirable goal. By leveraging the advantages of the As_2_O_3_/As(OH)_3_/P3MA nanocomposite, researchers aim to contribute to the advancement of both scientific understanding and practical applications in the field of photodetection and photodiodes.

The As_2_O_3_/As(OH)_3_/P3MA nanocomposite film operates through two steps, starting with the absorption of photons to generate hot electrons that transition to the upper level. These hot electrons then form clouds that collect on the surface of the inorganic materials, producing the J_ph_ values that are measured. Equation (3) [34] estimates the number of photons absorbed under full wavelength illumination, which is 8.0 × 10^21^ photons/s. By analyzing the J_ph_ values produced at various wavelengths, the effective photon number that produced the hot electrons can be estimated, which decreases from 8.0 × 10^21^ photons/s to 4.8 × 10^21^ photons/s from 340 to 540 nm. With the J_ph_ value increasing to 0.01 at 730 nm, the estimated effective photons become 5.6 × 10^21^ photons/s. The results show that the As_2_O_3_/As(OH)_3_/P3MA nanocomposite film can electroanalytically evaluate the wavelengths or intensities of incident photons with high sensitivity across a broad optical spectra from near IR to UV. Additionally, this estimation provides a way to evaluate the photodiode’s performance at these wavelengths.

(3) N=λP/hc

The operation of the photodiode device involves the interaction of light with the various layers and materials within the device structure. Each wavelength of light corresponds to a specific frequency and energy, which can have different effects on the photodiode. In the case of the n-layer As_2_O_3_/As(OH)_3_/P3MA, the incident light interacts with this layer, causing the generation of photoelectrons. These photoelectrons are created by the absorption of light energy, which promotes the production of photoactive electrons and holes.

The generated photoelectrons are collected on the surface of the n-layer. Due to the potential difference applied within the device [35], these photoelectrons can easily migrate toward the neighboring P-type layer (Ppy), which contains a higher concentration of holes. This migration of photoelectrons is driven by the potential gradient within the device.

Similarly, the holes in the P-type layer can also migrate under the influence of the applied potentials. These holes can move toward the n-type nanocomposite layer, which typically has a higher concentration of electrons. This movement of holes is facilitated by the potential difference within the device.

All of these transitions and movements of photoelectrons and holes are primarily motivated by the effect of incident light on the photodiode. The absorption of light and the subsequent generation of electron–hole pairs initiate a series of charge carrier movements within the device, enabling the detection and conversion of light into electrical signals. It is worth noting that the specific materials and device structure (As_2_O_3_/As(OH)_3_/P3MA nanocomposite and Ppy) illustrate unique properties and characteristics that contribute to the overall performance of the photodiode in response to light.

The sensitivity of the As_2_O_3_/As(OH)_3_/P3MA nanocomposite photoelectrode was estimated using the linear dynamic range (LDR) for the photoelectrode, which was calculated using Equation (4). The LDR value of 36 dB in the UV region indicates that the photoelectrode has a high photosensitivity and can accurately detect light intensities ranging from the minimum detectable level to 36 dB above that level without saturation or distortion. This value suggests that the photoelectrode is highly responsive to incident photons in the UV region and can provide a reliable and linear response across a wide range of light intensities.

Furthermore, this LDR value is promising because it implies that the photoelectrode can be synthesized using cost-effective and readily available materials. This suggests that the development of light detection systems based on such photoelectrodes can be cost-efficient while still offering excellent sensitivity and linearity. This suggests that the photoelectrode has great potential for use in electroanalytical light estimation for photodiode applications. In other words, the LDR value is a measure of the photoelectrode’s ability to detect and respond to light, and the high value obtained in this study indicates a promising application in photodiodes.

(4) $$\mathrm{LDR}=20.\log(\frac{J_{\mathrm{ph}\;}}{J_{o}})$$

**Figure 6:**
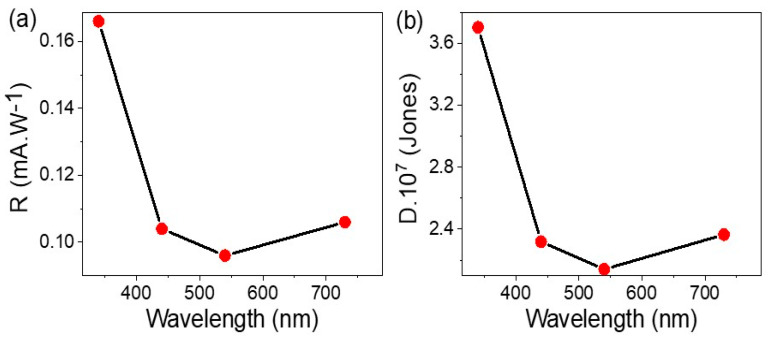
The study of the efficiency of the As_2_O_3_/As(OH)_3_/P3MA nanocomposite film through estimation of the (**a**) R and (**b**) D values under λ (340–730 nm).

Equation (5) [36] was used to estimate the sensitivity of the prepared As_2_O_3_/As(OH)_3_/P3MA nanocomposite photoelectrode by taking into account the R values, which convert incident photons into an electrical signal on the surface area of the photoelectrode (S). Figure 6a shows the R values under a wide optical region, where the optimum value of 0.16 mA·W^−1^ was observed at 340 nm, and it decreased to 0.10 mA·W^−1^ in the IR region. This high R value over a wide optical spectra, combined with the high photoresponsivity, suggests that this material or device is efficient at converting photons into electrical charge, making it desirable for applications such as photodetectors and photodiodes.

(5) $$\;R=\frac{J_{\mathrm{ph}}-{\;J}_{d}}{P.S}$$

The sensitivity of a photodetector or a photodiode to weak optical signals can be evaluated by calculating the D values at various wavelengths, as shown in Equation (6) and Figure 6b. Similarly, the fabricated As_2_O_3_/As(OH)_3_/P3MA nanocomposite photoelectrode exhibited high sensitivity in the UV region, with decreasing sensitivity at longer wavelengths. The optimal D value was 3.7 × 10^7^ Jones at 340 nm, while the smallest value was 2.4 × 10^7^ Jones at 730 nm. To further confirm the promising results of this photoelectrode and its potential for use in photodetectors and photodiodes for commercial applications in highly technological systems and devices, Table 1 compares these results with those of previous studies.

(6) $$D=R\;\sqrt{S\;/2{\;e\;J}_{o}\;}$$

## 4. Conclusions

This study described the synthesis of a nanocomposite photoelectrode by decorating poly-3-methyl aniline with As_2_O_3_/As(OH)_3_. The resulting material had a wide bandgap of 1.73 eV, a porous structure, and an average crystalline size of 22 nm. The photoelectrode showed promising electrical properties with a high sensitivity to different wavelengths, making it suitable for electroanalytical applications as a photodetector or photodiode. The values of J_ph_ and J_o_ were found to be 0.015 and 0.004 mA·cm^−2^, respectively, and the effective photon numbers were calculated to be 8.0 × 10^21^ and 5.6 × 10^21^ photons/s for wavelengths ranging from 340 to 730 nm. The R values were found to be 0.16 and 0.10 mA·W^−1^ for the same wavelength range. The high sensitivity of this nanocomposite material indicates its potential for use in highly technological systems and devices.

## Figures and Tables

**Table 1:** The study of the efficiency of the As_2_O_3_/As(OH)_3_/P3MA nanocomposite film through estimation of the definite wavelength R values.

Structure	Wavelength (nm)	Bais (V)	R (mA·W^−1^)
Graphene/P3HT [21]	325	1	NA
Polyaniline/MgZnO [16]	250	5	0.1
ZnO-CuO [20]	405	1	3 × 10^−3^
CuO/Si Nanowire [37]	405	0.2	3.8 × 10^−3^
PbI_2_-graphene [38]	550	2	NA
PbI_2_-5%Ag [39]	532	6	NA
GO/Cu_2_O [40]	300	2	0.5 × 10^−3^
ZnO/RGO [41]	350	5	1.3 × 10^−3^
ZnO/Cu_2_O [19]	350	2	4 × 10^−3^
TiN/TiO_2_ [42]	550	5	-
CuO nanowires [43]	390	5	-
ZnO/RGO [41]	350	5	1.3 × 10^−3^
As_2_O_3_/As(OH)_3_/P3MA (this work)	440	2	0.16

## Data Availability

All data generated or analyzed during this study are included in this article.
